# Magnetic Resonance Imaging (MRI) Findings in Pediatric Autoimmune Encephalitis: A Case Report

**DOI:** 10.7759/cureus.79453

**Published:** 2025-02-22

**Authors:** Amira E Raslan

**Affiliations:** 1 Radiology Department, Dallah Hospital, Riyadh, SAU

**Keywords:** autoimmune encephalitis, cerebrospinal fluid, diagnosis, magnetic resonance imaging, pediatrics

## Abstract

Autoimmune encephalitis (AE) is a rare condition that involves an immune-mediated attack on the brain, often presenting with seizures, altered consciousness, and other neuropsychiatric symptoms. This case report describes a seven-year-old girl who presented with acute brain dysfunction, including seizures and disturbed consciousness, following a gastrointestinal illness. Magnetic resonance imaging (MRI) findings revealed early signs of AE, including hyperintensities in the external capsules and medial temporal lobe. Following negative infectious workups, the diagnosis of AE was confirmed, and the patient was treated with immunomodulatory therapy, leading to gradual improvement. This case emphasizes the importance of early recognition, the role of MRI in diagnosis, and the need for prompt treatment to improve outcomes in pediatric AE.

## Introduction

Autoimmune encephalitis (AE) denotes a range of conditions marked by inflammatory processes that impact brain tissue, stemming from an immune-mediated pathogenic mechanism that targets neurons [1].

AE is characterized by an immune attack on neuronal antigens that results in the inflammation of the central nervous system, causing a spectrum of neuropsychiatric symptoms. In the pediatric population, headaches, seizures, movement disorders, and behavioral changes are the most common presenting symptoms of AE [2].

Magnetic resonance imaging (MRI) is a cornerstone in the diagnostic process of AE, providing valuable insights into the structural and functional changes associated with the condition. Specific imaging patterns, such as hyperintensities in the medial temporal lobe or other characteristic regions, help differentiate AE from other neurologic disorders. When combined with clinical findings and laboratory data, MRI can be pivotal in confirming the diagnosis and guiding management strategies [3,4].

This case report discusses the MRI findings in pediatric AE, emphasizing the role of imaging in the diagnostic process.

## Case presentation

A seven-year-old female patient, weighing 35 kg and measuring 150 cm, presented with acute brain insult characterized by disturbed consciousness, recurrent convulsions, and coma. The initial assessment found that her vital signs were within normal ranges, with a temperature of 36.5°C and blood pressure of 96/60 mmHg. The patient had a history of persistent vomiting, diarrhea, and poor feeding, which had not responded to home treatment. Despite initial management efforts, her symptoms persisted, prompting a need for further investigation due to suspected AE.

The laboratory profile indicated notable inflammatory markers and mild metabolic disturbances. C-reactive protein (CRP) levels initially spiked significantly, peaking at 116 mg/L. The complete blood count (CBC) showed neutrophilia at 63.7%, alongside a low hemoglobin level of 8.9 g/dL and hematocrit at 27.3%. Kidney function tests revealed minor fluctuations, with serum creatinine measured at 37 μmol/L and a transient, mild elevation in blood urea nitrogen (BUN). The liver profile revealed elevated enzymes, with alanine aminotransferase (ALT) at 75 U/L and aspartate aminotransferase (AST) at 73 U/L. There was a significant increase in gamma-glutamyl transferase (GGT) to 265 U/L (Table 1).

**Table 1:** Laboratory findings

Test	Result	Reference range
C-reactive protein	116 mg/L	<10 mg/L
Neutrophils	63.7%	40-60%
Hemoglobin	8.9 g/dL	11.5-15.5 g/dL
Hematocrit	27.3%	35-45%
Serum creatinine	37 μmol/L	27-62 μmol/L
Blood urea nitrogen	7 mmol/L	2.6-6 mmol/L
Alanine aminotransferase	75 U/L	7-56 U/L
Aspartate aminotransferase	73 U/L	10-40 U/L
Gamma-glutamyl transferase	265 U/L	12-43 U/L
Procalcitonin	<0.1 ng/mL	<0.5 ng/mL

Upon admission to the pediatric intensive care unit (PICU), the patient received comprehensive supportive care for her bed-bound status. Given her critical condition, mechanical ventilation was initiated via endotracheal intubation. She was successfully extubated and transitioned to nasal oxygen. A central line was placed to administer intravenous fluids (D5 NS), nasogastric tube (NGT) feeding, and necessary medications. The patient was started on meropenem for antibiotic coverage, and antiviral acyclovir was administered initially but discontinued after re-evaluation.

Antiepileptic management included valproate, phenytoin, and phenobarbitone, supplemented by midazolam and fentanyl infusions, both of which were eventually stopped. Clobazam was added following a neurology consultation due to persistent subclinical seizure activity, as confirmed by continuous electroencephalogram (EEG) monitoring. Immunomodulatory therapy was initiated with intravenous immunoglobulin (IVIG) at 1 g/kg, followed by high-dose pulse steroids at 30 mg/kg to address the suspected autoimmune etiology.

Laboratory findings also showed normal procalcitonin (PCT) levels, while hemoglobin remained stable. A thorough workup for autoimmune markers and infectious causes was negative, further strengthening the diagnosis of AE.

**Figure 1:**
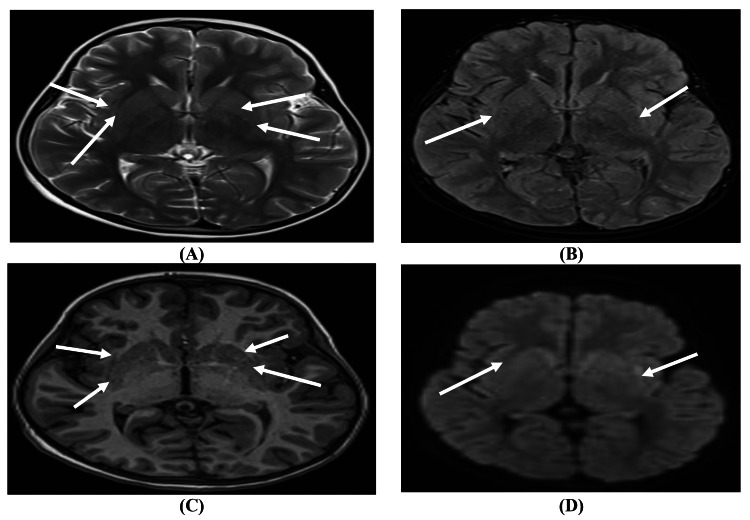
Characteristic findings of autoimmune encephalitis in a seven-year-old girl presenting with acute encephalopathy and recurrent seizures. (A) Axial T2-weighted imaging sequence. (B) Axial FLAIR sequence. (C) Axial T1 magnetization-prepared rapid acquisition gradient-echo sequence. (D) Axial DWI sequence showing bilateral external capsule/claustrum hyperintensities in T2/FLAIR with restriction in DWI T2/FLAIR: T2-weighted/fluid-attenuated inversion recovery; DWI: diffusion-weighted imaging

**Figure 2:**
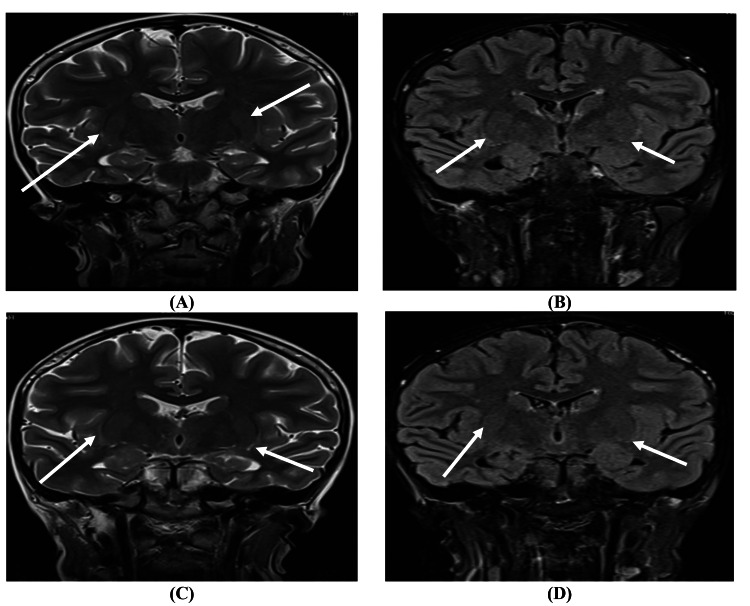
(A, C) Coronal T2-weighted imaging sequence. (B, D) Coronal FLAIR sequence showing bilateral external capsule/claustrum hyperintensities in coronal T2/FLAIR T2/FLAIR: T2-weighted/fluid-attenuated inversion recovery

An MRI of the brain with contrast was performed using MRI Siemens Magnetom Aera 1.5 T MRD001 (Siemens Healthineers, Erlangen, Germany), revealing notable findings. T2-weighted/fluid-attenuated inversion recovery (T2/FLAIR) imaging demonstrated hyperintensity in the bilateral external capsules with restricted diffusion on diffusion-weighted imaging (DWI). Additionally, a minimal faint hyperintensity was noted in the left medial temporal lobe on FLAIR and DWI, indicating early involvement, possibly due to AE. Posterior fossa findings showed an enlarged retrocerebellar space on the left, suggesting a small arachnoid cyst rather than an enlarged cisterna magna. No abnormalities were detected in the brainstem, cerebellum, or ventricles, and there was no evidence of space-occupying lesions or midline shift (Figure 1 and Figure 2).

Magnetic resonance venography (MRV) and magnetic resonance angiography (MRA) studies ruled out any major vascular abnormalities, with cerebral arteries and venous sinuses exhibiting normal flow and caliber. There was no evidence of venous thrombosis, arteriovenous malformation, or aneurysmal formation, providing further confirmation that the MRI findings were not vascular in nature.

The MRI findings, specifically the bilateral external capsule hyperintensities and subtle left medial temporal lobe changes, were consistent with early-stage AE. The absence of contrast enhancement and structural abnormalities suggested an inflammatory process rather than an ischemic or mass-related pathology. Additionally, negative serologic and infectious workup reinforced the clinical suspicion of an autoimmune etiology, likely triggered by her recent gastrointestinal illness.

The treatment plan, including immunosuppression with IVIG and pulse steroids, along with intensive supportive care, reflects current approaches to managing refractory AE in pediatric patients.

Following the initiation of treatment, the patient began to show gradual improvement. She was successfully weaned from oxygen support, and oral feeding was reintroduced with careful monitoring to prevent aspiration. Plans were made for extensive rehabilitation and physical reconditioning as part of her continued recovery and long-term care.

## Discussion

Early detection of AE is essential, as prompt diagnosis and the initiation of appropriate immunotherapy are essential for improving patient outcomes, minimizing the likelihood of long-term neurological complications, and enhancing quality of life. However, achieving a timely diagnosis is often challenging. The initial presentation of AE is typically nonspecific, and the condition's rarity contributes to diagnostic delays. Additionally, the clinical features of AE frequently overlap with those of infectious encephalitis, primary psychiatric disorders, and other autoimmune diseases, further complicating the diagnostic process. The diagnosis of AE currently depends on a multifaceted approach that integrates clinical presentation, neuroimaging findings, cerebrospinal fluid (CSF) analysis, and the identification of specific autoantibodies [5].

In this case, the MRI findings were pivotal in supporting the diagnosis of AE. The T2/FLAIR hyperintensities in the bilateral external capsules and subtle hyperintensity in the left medial temporal lobe are consistent with early-stage inflammatory processes characteristic of AE. Such findings align with established patterns observed in AE, where medial temporal lobe involvement is commonly reported due to its vulnerability to immune-mediated inflammation [6,7].

Also, Hartung et al. [8] stated that in AE, particularly with leucine-rich glioma-inactivated 1 (LGI1), contactin-associated protein-like 2 (CASPR2), or glutamic acid decarboxylase (GAD) antibodies, patients frequently exhibit unilateral or bilateral T2/FLAIR hyperintensities in the medial temporal lobe, which can progress to hippocampal atrophy. In contrast, Barbagallo et al. [9] reported that MRI findings are neither sensitive nor specific for AE. In children, FLAIR or T2 hyperintensities in medial temporal lobes, brainstem or, in some cases, subcortical regions, and cerebellum may be present. Gadolinium enhancement is variable.

The absence of contrast enhancement and lack of structural abnormalities, such as space-occupying lesions or midline shifts, helped exclude differential diagnoses like ischemia, tumor, or infectious causes [10]. Moreover, excluding vascular abnormalities via MRV and MRA provided further evidence against conditions such as venous thrombosis or arteriovenous malformations, solidifying the autoimmune basis of the encephalopathy [11].

The patient's clinical presentation, marked by altered consciousness, recurrent seizures, and a history of gastrointestinal illness, raises diagnostic challenges due to the overlap with other neurologic and systemic conditions. The negative serologic and infectious workup played a critical role in ruling out alternative causes, such as infectious encephalitis, thereby supporting the autoimmune hypothesis [12].

MRI facilitates the diagnosis of AE and serves as a tool for monitoring disease progression and treatment response. In this case, the findings of restricted diffusion and inflammation on DWI and FLAIR sequences indicated a potentially reversible inflammatory process in the absence of progressive changes or additional structural damage. This aligns with the clinical trajectory observed post-treatment, where the patient showed signs of gradual improvement [13].

## Conclusions

An integrated diagnostic strategy, including clinical presentation, neuroimaging, and laboratory data, is important to overcome the AE's vague and overlapping symptoms. MRI results guided prompt immunomodulatory treatment, including IVIG and high-dose steroids, which helped the patient recover. Pediatric AE results depend on early detection and treatment to reduce neurological sequelae and increase quality of life. MRI can monitor disease progression and therapy response.
